# A Novel Adaptive Recursive Least Squares Filter to Remove the Motion Artifact in Seismocardiography

**DOI:** 10.3390/s20061596

**Published:** 2020-03-13

**Authors:** Shuai Yu, Sheng Liu

**Affiliations:** 1 School of Mechanical Science and Engineering, Huazhong University of Science and Technology, 1037 Luoyu Road, Wuhan 430074, China; yushuai91@hust.edu.cn; 2 Key Lab for Hydropower Transients of Ministry of Education, School of Power and Mechanical Engineering, Wuhan University, 8 East Lake South Road, Wuhan 430072, China; 3 Institute of Technological Sciences, Wuhan University, 8 East Lake South Road, Wuhan 430072, China

**Keywords:** adaptive recursive least squares filter (ARLSF), Seismocardiography (SCG), motion artifact, Electrocardiogram (ECG), heart rate

## Abstract

This paper presents a novel adaptive recursive least squares filter (ARLSF) for motion artifact removal in the field of seismocardiography (SCG). This algorithm was tested with a consumer-grade accelerometer. This accelerometer was placed on the chest wall of 16 subjects whose ages ranged from 24 to 35 years. We recorded the SCG signal and the standard electrocardiogram (ECG) lead I signal by placing one electrode on the right arm (RA) and another on the left arm (LA) of the subjects. These subjects were asked to perform standing and walking movements on a treadmill. ARLSF was developed in MATLAB to process the collected SCG and ECG signals simultaneously. The SCG peaks and heart rate signals were extracted from the output of ARLSF. The results indicate a heartbeat detection accuracy of up to 98%. The heart rates estimated from SCG and ECG are similar under both standing and walking conditions. This observation shows that the proposed ARLSF could be an effective method to remove motion artifact from recorded SCG signals.

## 1. Introduction

Seismocardiography (SCG) is a non-invasive measurement that records the local vibrations of the chest wall in response to the heartbeat [1]. SCG was first discovered in 1961 [1], and its first clinical application was used 30 years later in 1991 [2]. With the development of an accelerometer with high-sensitivity, low-noise, small-size, high-efficiency, and high-robustness signal-processing technology, SCG has shown its great potential to be used by wearables. Consequently, it is now feasible to use the information in clinical applications [3,4]. 

However, there are still some limitations in SCG measurements and assessments. Motion artifact is one of the major limitations. As a result, SCG research on motion artifact has been very active in recent years. Motion artifact is usually irregular and it is mixed with the heartbeat signals in the time and frequency domains. The mixture makes it difficult to separate the heartbeat signal from the mixed signal [3,4,5]. Some researchers tried to use multiple sensors to remove the motion artifact from the recorded signals [6,7,8,9]. The tri-axis acceleration data collected from a chest-worn accelerometer were utilized to remove motion artifact in an electrocardiogram (ECG) signal in 2003 [6], 2008 [7] and 2010 [8]. An independent component analysis approach and a normalized least means square (NLMS) adaptive filter to motion artifact cancellation of the SCG signal using two accelerometers were developed in [9] and [10], respectively. In these studies, one accelerometer was placed at the center of the sternum and the other was attached to the right side of the back of the subjects. The results were promising, but multiple sensors used in the experiments increased the complexity of the SCG measurement and assessment.

Meanwhile, other researchers developed several algorithms to remove motion artifact from the SCG signal by using only one accelerometer [11,12,13,14,15,16,17]. An ensemble empirical mode decomposition method was developed to remove white noise from a synthetic vibrocardiographic signal in [11] and the same method was successfully utilized to reduce the motion artifacts generated due to walking at normal and moderately fast speeds at a treadmill [12]. However, SCG signal could not be well recovered from corrupted signal. Rienzo et al. utilized an accelerometer to record a 24-hour SCG signal from freely moving subjects [13]. A movement-free SCG was extracted from the recorded accelerometer data using a continuous 5-second segment-based method. The results were promising, but the physiological parameters were not extracted from the signal. Pandia et al. designed a Savitzky Golay-based polynomial smoothing algorithm to extract the primary heart sound from the accelerometer data during walking [14]. The primary heart sound detection rate was up to 99.36%, but the graph of the extracted SCG signal could not be recovered. In another approach, the motion-free SCG signal was successfully extracted using a time delay-based normalized least mean square (NLMS) adaptive filter [17]. However, an extra moving average method was utilized to obtain the heart rate as the primary heart sound graph was not clear in the extracted motion-free SCG signal.

To solve this problem, we present a novel adaptive recursive least squares filter (ARLSF) for motion artifact removal in the SCG signal that was obtained by one accelerometer. The primary heartbeat signal graph is very clear in the motion artifact removed SCG signal without any other signal-processing procedures. 

In Section 2 of this paper, the main idea of ARLSF is introduced and two major parameters of ARLSF are discussed. The measurement system including the hardware system, experimental setup and software system are discussed in Section 3. Section 4 shows the results and Section 5 concludes this paper.

## 2. Theory of Adaptive Recursive Least Squares Filter (ARLSF)

### 2.1. The Principle of Adaptive Recursive Least Squares Filter

**Figure 1:**
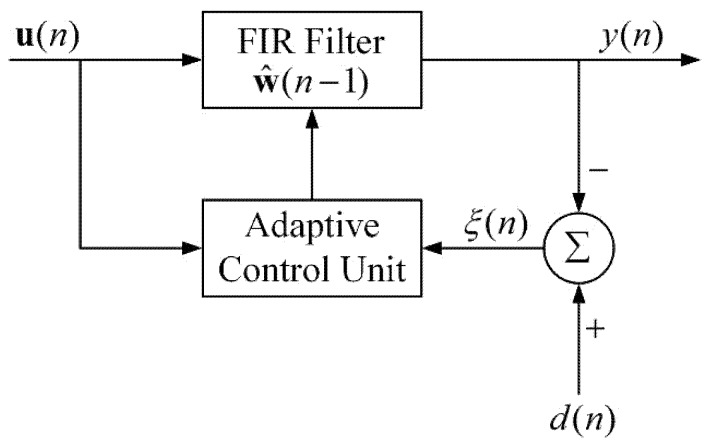
Block diagram of adaptive recursive least squares filter (ARLSF).

**Figure 2:**
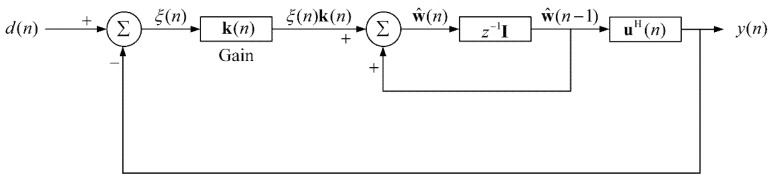
Signal-flow graph of ARLSF.

Figure 1 illustrates the block diagram of ARLSF. An RLS filter is a finite impulse response (FIR) filter of length M with coefficients w(n) [18,19]. The input vector u(n) is passed through the FIR filter to produce the output vector y(n). At each time-step, the coefficients are updated through the adaptive control unit using the input vector u(n)**.** The prior estimation error ξ(n) is described in Figure 2. All the parameters are defined in Equations (1)–(4) where d(n) is the desired signal.

(1) $$w(n)={\left[w_{0}(n),w_{1}(n),\cdots ,w_{M-1}(n)\right]}^{T}$$

(2) $$u(n)={\left[u(n),u(n-1),\cdots ,u(n-M+1)\right]}^{T}$$

(3) $$y(n)=w^{H}(n-1)u(n)$$

(4) ξ(n)=d(n)−y(n)

The adaptive control unit updates the coefficients using the input vector and the prior estimation error. The detailed information can be described in Equation (5):

(5) $$\hat{w}(n)=\hat{w}(n-1)+k(n)\xi (n)$$

where k(n) is the gain vector that is described in Equation (6):

(6) $$k(n)=\frac{\lambda^{-1}P(n-1)u(n)}{1+\lambda^{-1}u^{H}P(n-1)u(n)}$$

where λ is the forgetting factor and P(n) is a covariance matrix of the noise which can be updated by Equation (7):

(7) $$P(n)=\lambda^{-1}P(n-1)-\lambda^{-1}k(n)u(n)P(n-1)$$

### 2.2. Discussion of the Desired Signal

The design of the desired signal is very important in the RLS filter when the desired signal cannot be directly obtained from an aiding sensor. The RLS filter works under the premise that the desired signal is linearly correlated to the input signal and orthogonal to the estimation error. The RLS filter will perform better with the higher linear correlation and the stronger orthogonality mentioned above [18,19]. 

The desired signal and the estimation error are the motion artifact and the SCG signal, respectively. They are collected from a single accelerometer simultaneously and they are aliased in the frequency domain with different frequency characteristics. The collected acceleration data contains the heartbeat signal, the motion artifact, the respiratory component, the noise of the hardware system and sounds from the other organs. The frequency of the respiratory component is less than 1 Hz [20,21], and the frequency of the heartbeat signal can be up to 25 Hz [22,23]. As a result, the collected acceleration data is band-pass filtered from 1 to 25 Hz with a 32th order FIR filter to remove the gravity component, the respiratory component and the high frequency noise, and the filtered data composed of the expected SCG signal and motion artifact within a frequency range from 1 to 25 Hz can be set as the input of ARLSF.

**Figure 3:**
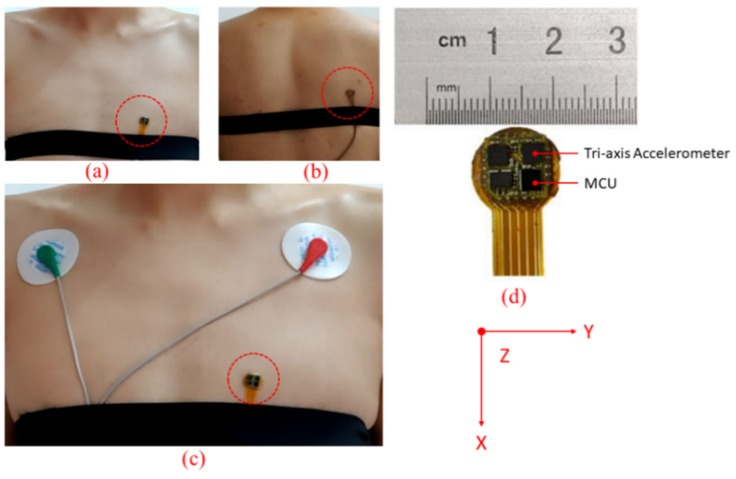
(**a**,**b**) Seismocardiography (SCG) recorder system placement for measuring heartbeat signal and motion signals, respectively. (**c**) A pair of electrocardiogram (ECG) lead I electrodes placement and SCG recorder system placement. (**d**) An image of the SCG recorder system which shows the dimensions. *X*-axis, *y*-axis and *z*-axis describe the head to foot, shoulder to shoulder and dorsoventral direction, respectively.

**Figure 4:**
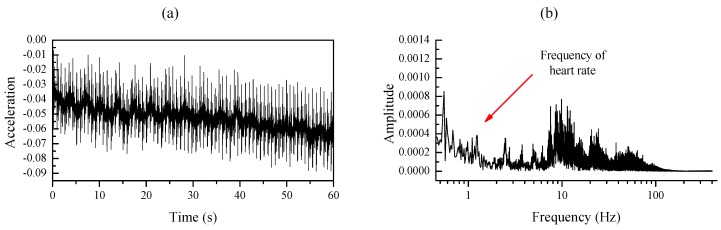
(**a**,**b**) Time plot and frequency plot of heartbeat signal respectively.

**Figure 5:**
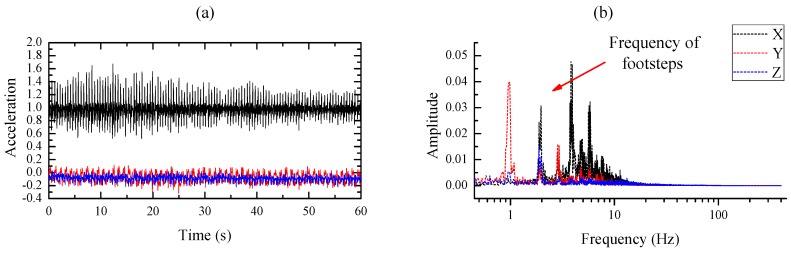
(**a**,**b**) Time plot and frequency plot of the tri-axis motion signal respectively.

In addition, the maximum heart rate of a healthy adult is less than 210 beats per minute (bpm) [24,25,26] which illustrate the maximum frequency of an adult’s heart rate is 3.5 Hz. In order to further analyze the frequency of the motion signal and the heartbeat signal, a similar experiment has been conducted to record the motion signal and the heartbeat signal [9]. Firstly, the SCG recorder system is attached to the chest wall of the subject to record the heartbeat signal under the condition of stand-up without moving as shown in Figure 3a; *z*-axis acceleration data is collected at a sampling rate of 800 Hz for about 120 s. Afterward, the same SCG recorder system is placed at the right side of the back of the same subject to record the motion artifact at a sampling rate of 800 Hz as shown in Figure 3b. The subject is asked to walk on a treadmill that works at a low speed (3–5 km/h) and the tri-axis acceleration data are collected for about 120 s. Four continuous 60-second signals are selected from the middle of each recorded acceleration data respectively and they are analyzed in time and frequency domain. Figure 4a illustrates that the heart rate frequency (1.2 Hz) lies in the low-frequency range(<3.5 Hz) while high frequency heart sound signal lies in the range from 4 Hz to 25 Hz. An obvious low-frequency point (1.82 Hz) representing the footsteps frequency is marked in Figure 5b, and the high-frequency component of the motion concentrates on the range from 4 Hz to 10 Hz [15,27]. It can be observed that the heartbeat signal and the motion signal are overlapped in the frequency domain and cannot be separated by bandpass filters.

**Figure 6:**
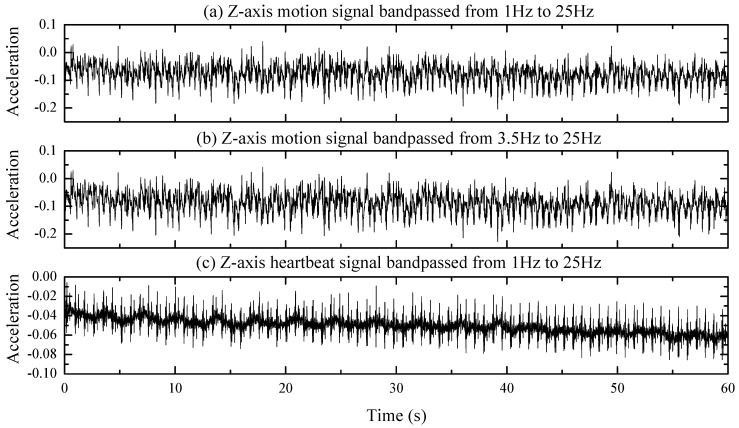
(**a**) *Z*-axis motion signal band passed from 1 to 25 Hz. (**b**) The *Z*-axis motion signal band passed from 3.5 to 25 Hz. (**c**) The *Z*-axis heartbeat signal band passed from 1 to 25 Hz.

Further process procedures including bandpass filtering and correlation analyzation are performed on the *z*-axis of the motion signal and the heartbeat data. The *z*-axis of the motion signal and heartbeat signal are bandpass filtered from 1 Hz to 25 Hz, and the filtered signals are plotted in Figure 6a,c respectively. Figure 6b represents the filtered signal of the *z*-axis of the motion signal through the FIR bandpass filter of the same type as Figure 6a,c, but at different cutoff frequencies from 3.5 Hz to 25 Hz. Meanwhile, the correlation coefficient between signals in Figure 6a,b is calculated to be 0.99978 and the counterpart between signals in Figure 6a,c is 0.15374, which proved well the high linear correlation of the signals in Figure 6a,b, and the strong orthogonality of the signals in Figure 6a,c [28]. Therefore, the desired signal can be obtained by bandpass filtering the recorded acceleration signal from 3.5 to 25 Hz. 

### 2.3. Discussion of the Forgetting Factor

The forgetting factor plays an important role in the behavior of the RLS algorithm under non-stationary conditions. In a classical RLS algorithm, the value of the forgetting factor is fixed between 0 and 1. For the value of the forgetting factor closer to 0, the RLS algorithm has not only a smaller memory length and fast-tracking ability but also a reduced convergence speed and stability. On the other hand, when the forgetting factor is closer to 1, the RLS algorithm has fast convergence and good stability, but the tracking ability suffers and the memory length becomes longer [18,19]. In order to meet the conflicting requirements in non-stationary conditions, the forgetting factor is set between 0.98 and 1.0 [29,30]. In theory, the optimal value of the forgetting factor in non-stationary conditions can be defined by Equation (8) [19]:

(8) $$\lambda \approx 1-\frac{1}{\sigma_{\nu }}{\left(\frac{tr[R_{\omega }]}{tr[R_{u}^{-1}]}\right)}^{1/2}$$

where $\sigma_{\nu }^{2}$, $R_{\omega }$, $R_{u}$ are the measurement noise variance, process noise correlation matrix, and input vector correlation matrix respectively, tr[·] denotes the trace of the matrix.

## 3. Measurement Technique

### 3.1. Hardware System

Figure 3d shows the prototype of the SCG Recorder System (SRS), which integrates a commercial tri-axis accelerometer (ICM-20602 manufactured by InvenSense) and a microprocessor (STM32F411CEY6 manufactured by STMicroelectronics). The size of the device is less than 1 cm^2^. The tri-axis accelerometer is used to capture acceleration data including SCG signal and motion information within a range of ±2g. The micro controller unit (MCU) collects the acceleration data from the accelerometer via a serial peripheral interface (SPI) at a rate of 800 Hz. The SRS is attached to the chest wall and placed at the left of the sternum as shown in Figure 3c. The *z*-axis of SRS describes the dorsoventral direction of the subject, and the *x*-axis and *y*-axis describe the head to foot direction and the shoulder to shoulder direction respectively. 

In addition to the acceleration data captured by the tri-axis accelerometer, a standard ECG system simultaneously collects a standard ECG lead I signal at a rate of 512 Hz. The two electrodes are placed at the right arm and the left arm, respectively, as shown in Figure 3c. Both the SCG Recorder System and ECG system are connected to a host PC via serial cables for data transmission and synchronization.

### 3.2. Experiment Setup

The hardware system described above was used on sixteen subjects whose ages ranged from 24 to 35. The experiment was conducted on a treadmill and the subjects were asked to keep standing for at least 120 s before walking at least 180 s. After walking, the subjects stood for at least 60 s. The subjects could breathe freely during the whole experiment. The walking speed of the subjects was limited to less than 1.5 m/s by setting the treadmill at a low speed (3–5 km/h). The SCG and ECG signals including sampling time and sensor data were collected and transmitted to the PC for further analyzation in MATLAB (R2016a). 

### 3.3. Software System

MATLAB (R2016a) was used to analyze all the data. The processing procedure consists of three parts: signal preprocessing to obtain the primary and reference channel of the ARLSF, the ARLSF and feature extraction. 

#### 3.3.1. Signal Preprocessing

The collected acceleration data is a mixed collection of signals in both the time and frequency domains, which contain the heartbeat signal, the motion artifact, the respiratory component, the noise of the hardware system and sounds from the other organs. 

Firstly, the collected acceleration data is bandpass filtered from 1 to 25 Hz with a 32^th^ order FIR filter to remove the gravity component, the respiratory component and the high-frequency noise. This procedure leaves the expected SCG signal and motion artifact within a frequency range from 1 to 25 Hz in the filtered signal, which is fed to the primary channel of the ARLSF. 

**Figure 7:**
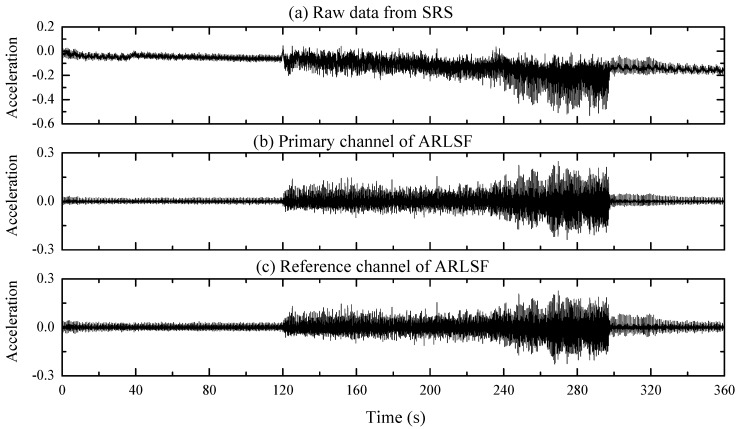
(**a**) Raw data from SCG Recorder System (SRS). (**b**,**c**) The primary and reference channel of ARLSF respectively.

The second filtered signal is obtained by bandpass filtering the acceleration data from 3.5 to 25 Hz with the same order and type of FIR filter. The correlation coefficient between the filtered signal of the primary channel and the second filter signal is calculated to be 0.987 which proved well the high linear correlation of these two filtered signals as discussed in Section 2.2. Therefore, the second filtered signal can be fed into the reference channel of the ARLSF. The raw data of the collected acceleration data, the primary channel obtained, and the reference channel are plotted in Figure 7a–c, respectively. 

#### 3.3.2. ARLSF

The same order and same type of FIR filter used in signal preprocessing section creates the same delay to both the primary channel and the reference channel. As a result, the primary channel and the reference channel are synchronized in the time domain. In addition, the forgetting factor is calculated to be 0.9908 based on Equation (8). The variable ξ(n) described in Equation (4) is the estimated heartbeat signal. 

#### 3.3.3. Feature Extraction

Features extracted from the ECG signal and filtered SCG signal are R peaks and aortic valve opening (AO) peaks, respectively. R peaks can be extracted from the ECG signal using the classical Pan Tompkin algorithm [31], while the extraction algorithm for AO peaks is different. The formula is described in Equations (9) and (10):

(9) [loc_min,val_min]=min(ξ(t−0.3:t))

(10) [loc_max,val_max]=max(ξ(t−0.3:t))

The estimated heartbeat signal ξ(n) is divided into several segments with a length of 0.3s [32]. ξ(t−0.3:t) is the segment at time t and loc_min, val_min, loc_max and val_max are the timestamp of the minimum value, the minimum value, the timestamp of maximum value and the maximum value of the segment at time t respectively. In addition, some constraints are used to avoid incorrect extracted AO peaks:

(11) $$\left\{ \begin{array}{l} val\_min<\;-0.007\\ val\_max\;>\;0.01\;\\ \left|loc\_max-loc\_min\right|<0.02 \end{array}\right.$$

**Figure 8:**
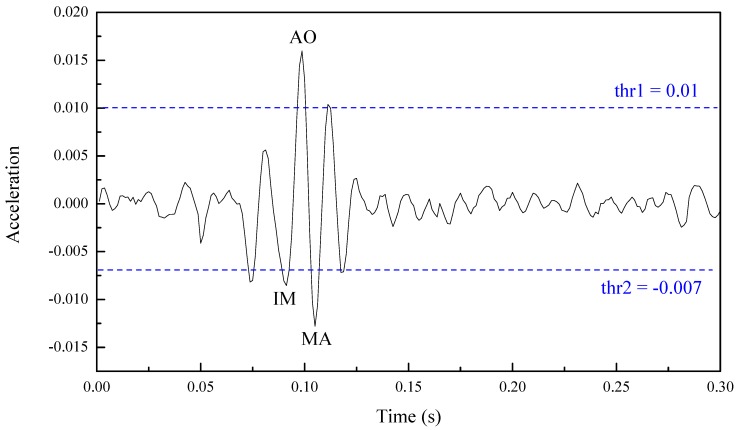
A correct aortic valve opening (AO) peak from a continuous 0.3s segment.

A continuous 0.3 s segment including a correct AO peak is picked from the estimated heartbeat signal and plotted in Figure 8. val_max represents the magnitude of the AO peak and val_min represents the magnitude of the successive isovolumic moment (IM) peak or maximum acceleration (MA) peak. Constant values -0.007 and 0.01 in Equation (11) are the maximum amplitude of the IM envelope peaks and minimum amplitude of the AO envelope peaks respectively [32]. In addition, the time interval between val_max and val_min should be restricted shorter than 0.02s [33]. When loc_min, val_min, loc_max and val_max meet the restrictions in Equation (11), loc_max and val_max are the timestamp and the value of the extracted AO peak respectively. For further analysis, the heart rate can be calculated from the R–R and AO–AO interval. 

## 4. Results

**Figure 9:**
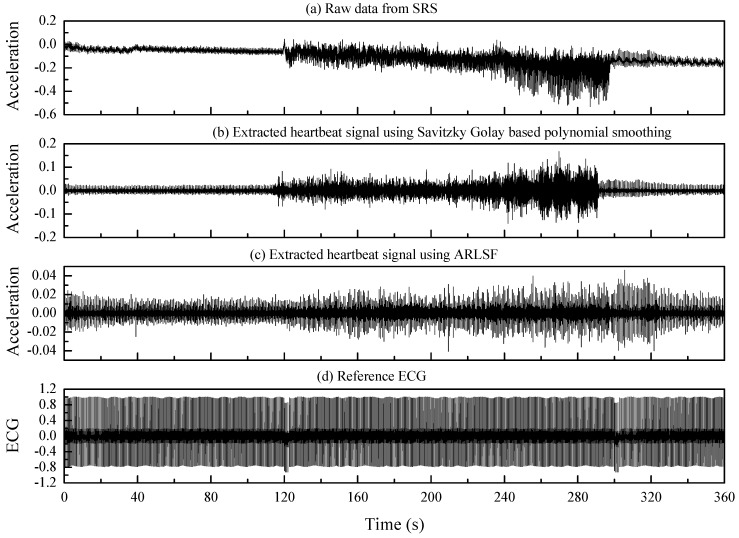
(**a**) Raw data from SRS. (**b**) Extracted heartbeat signal using Savitzky Golay filter. (**c**) Extracted heartbeat signal using ARLSF. (**d**) The reference ECG lead I signal. (The units of acceleration and ECG are g and mV, respectively.)

**Figure 10:**
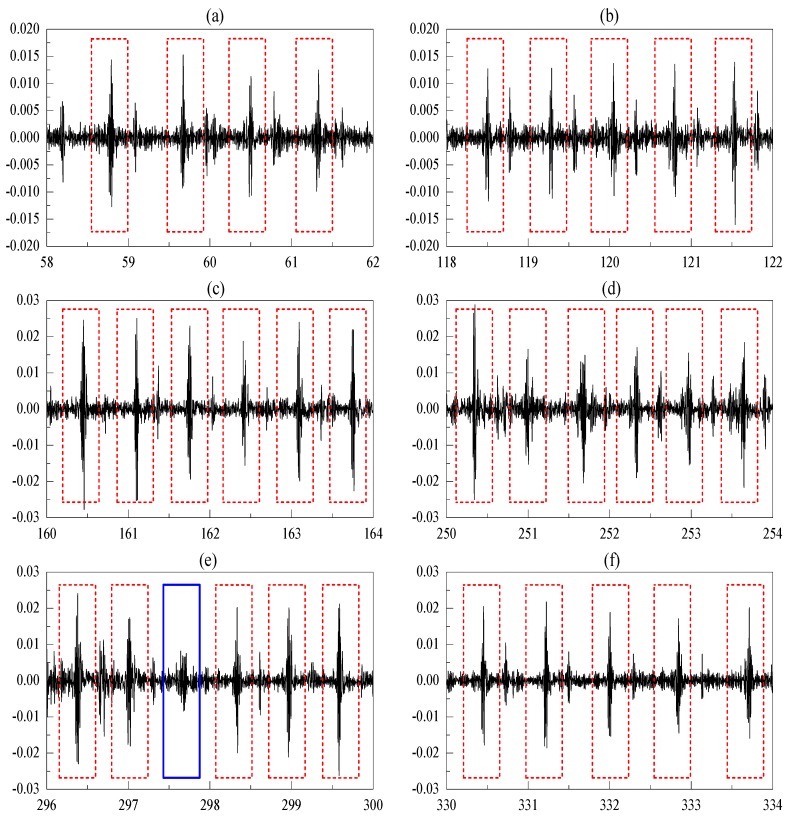
(**a**–**f**) Segmented signal during standing before walking, transitions from standing to walking, the first half of walking, the second half of walking, transitions from walking to standing, standing after walking. (The *x*-axis and *y*-axis are time in seconds and acceleration in gravity respectively.)

Figure 9 displays the raw data and processing results. Figure 9a shows the raw acceleration data collected from the SRS. The data show that the heartbeat signals are contaminated by the motion artifact, and the features and graphs of the heartbeat signals cannot be identified during the walking period. Figure 9b,c show the extracted heartbeat signals using Savitzky Golay-based polynomial smoothing [14] and ARLSF. Features and graphs of heartbeat signals are not clear from 120 s to 300 s in Figure 9b. It can be observed that the proposed ARLSF outperforms the Savitzky Golay-based polynomial smoothing. For better visualization, six segmented signals selected from the standing period before walking, transitions from standing to walking, the first half of walking, the second half of walking, transitions from walking to standing, and standing after walking are plotted in Figure 10a–f respectively. It is obvious that the features and graphs of the heartbeat signals are visually noticeable after ARLSF. Thus, any other signal-processing procedures are unnecessary on the heartbeat signals. 

### 4.1. Heartbeat Detection Accuracy

**Figure 11:**
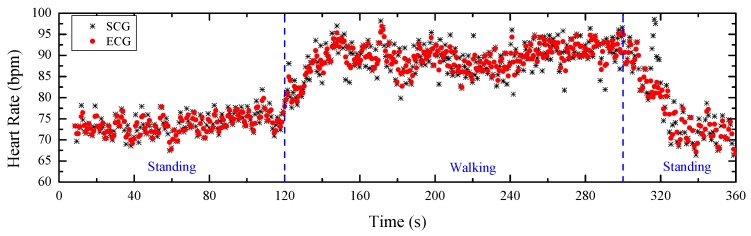
Heart rates estimated from ECG and SCG.

The standard ECG lead I recordings are the baseline references for heartbeat signals. Heartbeat detection accuracy is defined as the ratio of the detected SCG peaks divided by the detected ECG peaks. The detected SCG peaks are visually noticeable and marked with the red dotted line in Figure 11. In addition, the missing SCG peaks are also considered to give a more precise estimate of detection accuracy. These peaks represent the undetected and false-positive detected SCG peaks. The blue rectangular, which is marked in Figure 10, can be considered as an undetected SCG peak using the rule of feature extraction described previously. This method is used even though the features and graphs of the signal are visually noticeable. Numbers in the brackets represent the missing peaks that occur at the beginning and end of walking in Table 1. It can be observed that above 60% of the missing SCG peaks occur at the beginning and end of walking when MA changes rapidly. The detection rates, which are shown in Table 1, are higher than 98%.

### 4.2. Heart Rate Estimations

**Figure 12:**
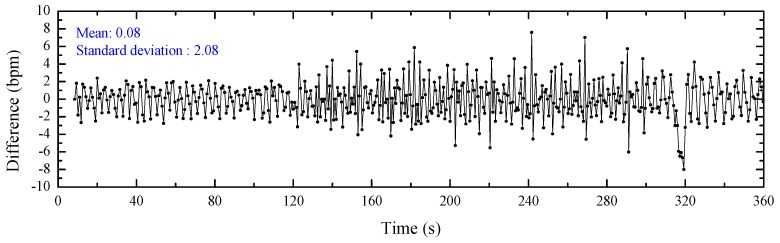
Heart rate difference between ECG and SCG.

The detected SCG peaks and ECG peaks evaluate the heartbeat signals from the perspective of the signal graph without considering the correctness. Heart rate can be an effective factor to verify the correctness of the detected peaks. As a gold standard in the clinical field, the heart rate estimated from the detected ECG peaks can be considered as the reference for the ground truth. Figure 11 illustrates the heart rates estimated from the detected SCG peaks and ECG peaks. These peaks are marked as black stars and red points, respectively. The *x*-axis represents the time that can be divided into three regions: standing, walking and return to standing. It can be observed that the heart rate is stable and low while standing, increases and then stabilizes at a relatively high level during walking, and then decreases to a stable and similar low magnitude when the subject stands again. The detailed difference between the heart rates from ECG and SCG with a mean value of 0.08 bpm and a standard deviation value of 2.08 bpm is plotted in Figure 12. It can be observed that the heart rates estimated from the detected SCG peaks and ECG peaks match very well.

### 4.3. Bland–Altman Analyzation

**Figure 13:**
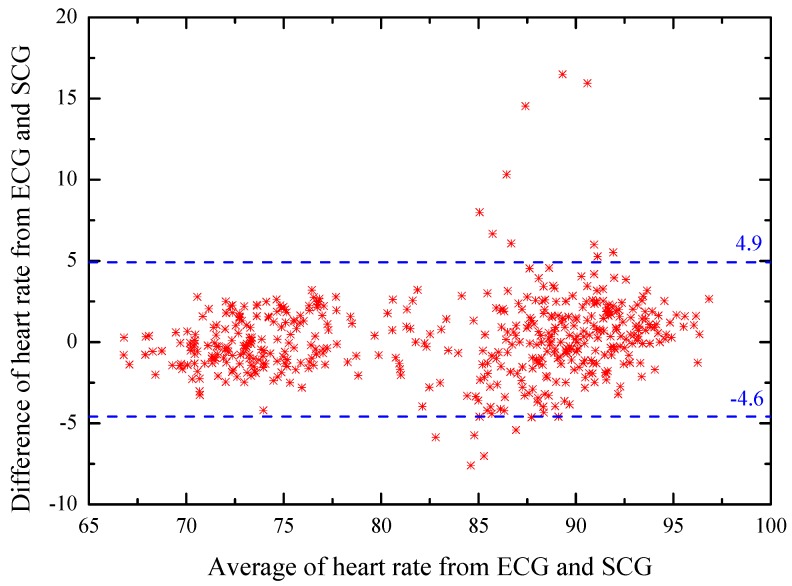
Bland–Altman plot of the heart rate measurements from ECG and SCG.

To further analyze the agreement between the heart rates estimated from the detected SCG peaks and ECG peaks, a Bland–Altman plot [34] is used and shown in Figure 13. Based on the definition of a Bland–Altman plot, the *x*-axis represents the average heart rates from SCG and ECG, and the *y*-axis represents their differences. A 95% confidence region is marked with blue dashed lines that have an upper threshold of 4.9 and a lower threshold of –4.6. It is observed that there are a few outliers, but overall most measurements lie in the 95% confidence region. 

## 5. Discussion and Conclusions

In this paper, we proposed a novel method based on an adaptive recursive least squares filter to remove the motion artifact of the acceleration data recorded by only one accelerometer. The primary channel of ARLSF containing the heartbeat signal and motion artifact signal within a frequency range of 1 to 25 Hz was obtained by bandpass filtering the recorded acceleration data from 1Hz to 25Hz. Then the same acceleration data was bandpass filtered from 3.5 to 25 Hz to remove the heart rate frequency and footsteps frequency component from the heartbeat and motion artifact signals, respectively. The filtered signal and the signal of the primary channel were proved to have high correlation, and the heartbeat and motion artifact signal were proved to have strong orthogonality, which proved well that the filtered data could be fed into the reference channel of the ARLSF. The heartbeat signal graph of the extracted SCG signal was very clear without the need for any other signal recovery procedures. The heartbeat detection accuracy was up to 98% and heart rates estimated from the SCG and ECG matched well under both the standing and walking conditions. 

At present these results are limited by a particularly rapidly changing motion artifact which will lead to missing SCG peaks’ detection. The forgetting factor is set at a fixed value at a balance of the convergency, stability and tracking ability which leads to good convergency and stability but poor tracking ability of the proposed ARLSF. Moreover, the forgetting factor calculated by Equation (8) was a globally optimal solution but not a locally optimal solution especially when motion artifact changes rapidly. To obtain better performance, future work on the ARLSF proposed in this paper will focus on the optimization of the forgetting factor by using an adaptive method. That method will change the forgetting factor in real time according to the changes of the motion and promote the performance of ARLSF. 

In addition, the experimental setup will be improved in future work. More subjects with a wider range of age and different health conditions, and more dynamic conditions including jumping and running, will be considered to evaluate the filter performance. In order to simplify the sensor placement before experiment and sensor data collection during the experiment, a miniature system-integrated SCG sensor, ECG sensor, MCU-embedded real-time filter algorithm and Bluetooth module will be developed in the future.

## Figures and Tables

**Table 1:** Heartbeat detection accuracy.

Subject No.	ECG Peaks Detected	SCG Peaks Detected	SCG Peaks Missing	Accuracy
1	483	478	5(2)	98.9%
2	475	468	7(4)	98.5%
3	472	468	4(3)	99.1%
4	480	475	5(3)	98.9%
5	488	483	5(3)	98.9%
6	478	473	5(2)	98.9%
7	492	483	9(6)	98.1%
8	501	496	5(2)	99.0%
9	495	490	5(2)	98.9%
10	490	486	4(1)	99.1%
11	477	471	6(5)	98.7%
12	486	483	3(2)	99.3%
13	496	491	5(3)	98.9%
14	491	489	2(2)	99.5%
15	485	477	8(4)	98.3%
16	482	474	8(6)	98.3%
